# Voice Analysis of Free-Speech for the Detection of Depression among Arabic-Speaking Refugee Women: A Pilot Machine Learning Study

**DOI:** 10.1192/j.eurpsy.2026.10594

**Published:** 2026-07-31

**Authors:** S. Özden, Z. Cömert, S. Gıca, Y. A. Acar, M. Ak

**Affiliations:** 1 Department of Psychiatry, Kızıltepe State Hospital, Mardin; 2 Department of Software Engineering, Faculty of Engineering and Natural Sciences, Samsun University, Samsun; 3 Department of Psychiatry, Faculty of Medicine, Necmettin Erbakan University, Konya, Türkiye; 4 Department of Pathology and Clinical Sciences Education, School of Medicine, Mercer University, Macon, GA, United States

## Abstract

**Introduction:**

Forced migration is a traumatic experience that heightens vulnerability to depression, with refugee women being particularly susceptible due to multiple stressors. Psychiatric assessment in this population is often constrained by language and cultural barriers, underscoring the need for accessible diagnostic tools. Voice analysis offers a promising approach, as depression is associated with measurable changes in acoustic features such as pitch, intensity, and timing.

**Objectives:**

To evaluate whether acoustic analysis of free speech can distinguish depressed refugee women from healthy controls, and to explore the potential of voice-based models as tools for mental health assessment of migrants. This pilot study also aimed to generate early evidence to guide future large-scale and validated research.

**Methods:**

Thirty-second free speech samples were recorded from 72 Arabic-speaking refugee women, including 31 with depression and 41 healthy controls, all aged 18–65 years and not using psychiatric medication. Recordings were acquired at 16 kHz and pre-processed with silence removal and data augmentation. The acoustic parameters included energy measures, spectral features (spectral centroid, bandwidth, roll-off, flatness, contrast), prosodic markers (pitch, voiced frame ratio, tempo), Mel-Frequency Cepstral Coefficients with derivatives, and chroma features. High-level embeddings were obtained using YAMNet and combined with acoustic features through feature-level fusion. Classification was conducted using support vector machines with radial basis kernel and XGBoost classifiers. Model performance was assessed with 10-fold cross-validation, reporting accuracy, recall, precision, F1 score, ROC-AUC, and precision–recall metrics.

**Results:**

The ensemble model achieved an accuracy of 0.594 (±0.174), precision of 0.618 (±0.135), recall of 0.755 (±0.204), and F1 score of 0.674 (±0.149). The overall discrimination performance was reflected by an ROC-AUC of 0.599 (±0.195) and an average precision of 0.726 (±0.127), based on 10-fold cross-validation.

**Conclusions:**

This pilot study focuses on Arabic-speaking refugee women, a population that is difficult to reach and relatively underrepresented in mental health research, and uses short free speech samples for the detection of depression. The findings highlight the feasibility and potential of voice-based assessment, which is non-invasive and scalable and can potentially overcome cultural and linguistic barriers inherent to traditional approaches. Nonetheless, our findings are preliminary, and larger, externally validated datasets, potentially incorporating multimodal features, are required prior to any clinical application.

**Disclosure of Interest:**

None Declared

